# Denervation Drives YAP/TAZ Activation in Muscular Fibro/Adipogenic Progenitors

**DOI:** 10.3390/ijms24065585

**Published:** 2023-03-15

**Authors:** Felipe S. Gallardo, Adriana Córdova-Casanova, Alexia Bock-Pereda, Daniela L. Rebolledo, Andrea Ravasio, Juan Carlos Casar, Enrique Brandan

**Affiliations:** 1 Departamento de Biología Celular y Molecular, Facultad de Ciencias Biológicas, Pontificia Universidad Católica de Chile, Santiago 7820436, Chile; 2 Centro Científico y Tecnológico de Excelencia Ciencia & Vida, Santiago 7750000, Chile; 3 Centro de Excelencia en Biomedicina de Magallanes (CEBIMA), Universidad de Magallanes, Punta Arenas 6213515, Chile; 4 Institute for Biological and Medical Engineering, School of Engineering, Medicine and Biological Sciences, Pontificia Universidad Católica de Chile, Santiago 7820436, Chile; 5 Departamento de Neurología, Pontificia Universidad Católica de Chile, Santiago 7820436, Chile; 6 Facultad de Medicina y Ciencia, Universidad San Sebastián, Santiago 7510602, Chile

**Keywords:** denervation, skeletal muscle, YAP/TAZ, FAPs, fibrosis

## Abstract

Loss of motoneuron innervation (denervation) is a hallmark of neurodegeneration and aging of the skeletal muscle. Denervation induces fibrosis, a response attributed to the activation and expansion of resident fibro/adipogenic progenitors (FAPs), i.e., multipotent stromal cells with myofibroblast potential. Using in vivo and in silico approaches, we revealed FAPs as a novel cell population that activates the transcriptional coregulators YAP/TAZ in response to skeletal muscle denervation. Here, we found that denervation induces the expression and transcriptional activity of YAP/TAZ in whole muscle lysates. Using the *Pdgfra*^H2B:EGFP/+^ transgenic reporter mice to trace FAPs, we demonstrated that denervation leads to increased YAP expression that accumulates within FAPs nuclei. Consistently, re-analysis of published single-nucleus RNA sequencing (snRNA-seq) data indicates that FAPs from denervated muscles have a higher YAP/TAZ signature level than control FAPs. Thus, our work provides the foundations to address the functional role of YAP/TAZ in FAPs in a neurogenic pathological context, which could be applied to develop novel therapeutic approaches for the treatment of muscle disorders triggered by motoneuron degeneration.

## 1. Introduction

Excessive and pathological deposition of skeletal muscle extracellular matrix (ECM)—*fibrosis*—defines one of the most common outcomes of many chronic skeletal muscle pathologies [1,2]. Degeneration of the neuromuscular junction (NMJ), which can be experimentally induced by the transection of motor nerves, precedes muscular fibrosis. This process occurs during aging and neurodegenerative diseases, such as amyotrophic lateral sclerosis (ALS), leading to muscle atrophy and decreased muscle performance [3]. Although crucial signaling pathways, such as the signaling mediated by the transforming growth factor type β1 (TGF-β1) [4,5] and the bioactive lipid lysophosphatidic acid (LPA) [6], have been described as promoters of skeletal muscle fibrosis, their molecular mediators are still poorly understood. Thus, discovering downstream molecules that transduce these signals may be relevant for the development of novel therapies that target skeletal muscle fibrosis and neurogenic degeneration.

Myofibroblasts are the primary cell type responsible for tissue fibrosis across organs [7]. In the skeletal muscle, these cells are mainly derived from the differentiation of a resident multipotent stromal cell population, also known as fibro/adipogenic progenitors (FAPs), which express the mesodermal markers PDGFRα and TCF4 (*Tcf7l2*) [8,9,10,11,12,13,14]. Activated FAPs accumulate in denervated skeletal muscles and skeletal muscles affected by ALS [15,16,17]. However, the molecular mechanisms that regulate FAPs behavior are far from being understood. In this context, Yes-associated protein 1 (YAP) and transcriptional co-activator with PDZ-binding motif (TAZ) have been shown to participate in the fibrogenic process in various organs, including kidney, lung, and liver [18,19,20,21].

YAP and TAZ (referred together as YAP/TAZ) are paralogs transcriptional coregulators that orchestrate several biological processes, such as stem cell specification, differentiation and proliferation, and organ size control [22]. These proteins act as co-activators of the transcriptional enhancer factor (TEA)-domain (TEAD1-4) family of transcription factors to control the expression of their target genes, such as *Ankrd1*, *Cyr61/Ccn1*, and the pro-fibrotic factor *Ctgf/Ccn2* [23,24]. Although they were initially discovered as final effectors of the Hippo signaling pathway, which modulates their phosphorylation status through the action of a serine kinase relay module, their participation in other pathways has been documented, revealing the complex nature of YAP/TAZ activity and proteostasis [22,25]. For instance, YAP/TAZ act in concert with the canonical TGF-β1–SMAD signaling [21,26,27], are involved in the destruction complex of WNT/β-catenin signaling [28,29], and participate as downstream effectors of the LPA–LPA receptors–G-proteins signaling [30]. In addition to biochemical regulation, YAP/TAZ behave as mechanosensors, shuttling between the nucleus and cytoplasm in response to extra- and intracellular forces [31]. For instance, high ECM stiffness and consequent increase in actomyosin tension promote the nuclear accumulation and activity of YAP/TAZ, whereas cells in soft extracellular environment show cytoplasmic localization and low TEAD-dependent transcription [32,33,34].

In the skeletal muscle, YAP/TAZ have been described as regulators of muscle stem cell (satellite cell) function and muscle mass [35,36]. Only one report has demonstrated that skeletal muscle denervation (by peroneal nerve transection) upregulates the expression of YAP in whole muscle and enhances its localization in myonuclei, a response related to the control of skeletal muscle atrophy [37]. The participation of other cell types residing in the skeletal muscle in the denervation process, such as FAPs, has not been addressed.

Using an experimental model of neurogenic degeneration of limb skeletal muscle (sciatic nerve transection) combined with transcriptomics analyses, we report that YAP and TAZ accumulate in atrophic-fibrotic denervated skeletal muscles and that FAPs represent one of the cell types where YAP/TAZ are activated. Our study establishes a fundamental groundwork to determine the function of YAP/TAZ during skeletal muscle fibrosis.

## 2. Results

### 2.1. YAP and TAZ Are Increased in Denervated Skeletal Muscles

**Figure 1:**
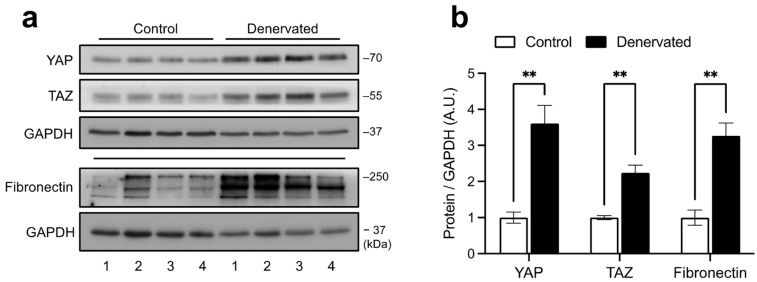
YAP/TAZ protein levels are increased in denervated skeletal muscles. (**a**) Western blot showing YAP/TAZ and fibronectin protein expressions in denervated and control (14 days post-denervation) TA muscles. Fibronectin and GAPDH were used as a fibrotic marker and loading control, respectively. For fibronectin, a second blot was carried out with the same samples. The numbers at the bottom indicate each independent mouse. (**b**) Densitometric analysis of YAP/TAZ and fibronectin signals normalized to their respective GAPDH. A.U. = arbitrary units. N = 4 independent mice. ** = *p* < 0.01; unpaired *t* test.

To understand the dynamics of YAP/TAZ activity in the skeletal muscle, we performed skeletal muscle denervation by sciatic nerve transection, a well-established muscle wasting and degeneration model. Limb muscles lacking motoneuron innervation undergo atrophy, FAPs expansion, and fibrosis, which becomes evident 14 days post-surgery [16,17]. Using this approach, we found that in whole tibial anterior (TA) muscle lysates from denervated muscles, both YAP and TAZ protein levels are increased compared to control muscles. Such increase was associated with fibrosis illustrated by the elevated ECM component fibronectin (Figure 1).

**Figure 2:**
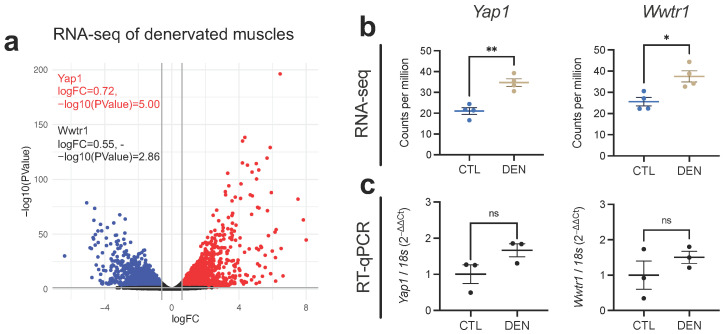
*Yap1* and *Wwtr1* mRNA levels are increased in denervated skeletal muscles. (**a**) Volcano plot showing differential expression analysis of RNA-seq data of denervated (DEN) vs. control (CTL) GST muscles. Values for *Yap1* and *Wwtr1* are highlighted. (**b**) RNA-seq analysis (counts per million of mapped reads) of *Yap1* and *Wwtr1* expressions from DEN and CTL muscles as in (**a**). N = 4 independent mice. * = *p* < 0.05, ** = *p* < 0.01; unpaired *t* test. (**c**) RT-qPCR analysis of *Yap1* and *Wwtr1* expression in DEN (14 days) and CTL soleus muscles. N = 3 independent mice. ns = not significant; unpaired *t* test.

To explore whether YAP/TAZ protein increase could be explained by its induction at the mRNA level, we processed and analyzed publicly available bulk RNA-sequencing data from mice gastrocnemius (GST) skeletal muscle, either denervated for 14 days or its control (SRA: SRP196460) [38]. Differential expression analysis using a *p* value of 0.05 and log2FC of 0.6 as thresholds revealed that of 29,327 mapped genes, 3121 were upregulated, 2683 downregulated, and 23,523 were not affected 14 days after denervation. The volcano plot shows that *Yap1* (log2FC = 0.72, *p* = 9.86 × 10^−6^) can be found among the upregulated genes. Although not included in the upregulated group, *Wwtr1* (TAZ) expression was significantly induced with a lower fold-change (log2FC = 0.55, *p* = 1.37 × 10^−3^) (Figure 2a). Replotting of the counts per million values from the differential expression analysis (TMM method) demonstrates increased expression of both genes (Figure 2b). The results from the RNA-seq expression analysis were further supported by RT-qPCR (Figure 2c). Despite the high variability in the data, RT-qPCR analysis demonstrated a similar upward trend in both *Yap1* and *Wwtr1* mRNA levels, which at least partially supports the RNA-seq findings. This additional validation using RT-qPCR strengthens the evidence of YAP and TAZ induction at the protein level in response to denervation and strongly suggests that an increase in their mRNA levels may partly explain this.

### 2.2. YAP/TAZ Activity Is Augmented in Denervated Skeletal Muscle

**Figure 3:**
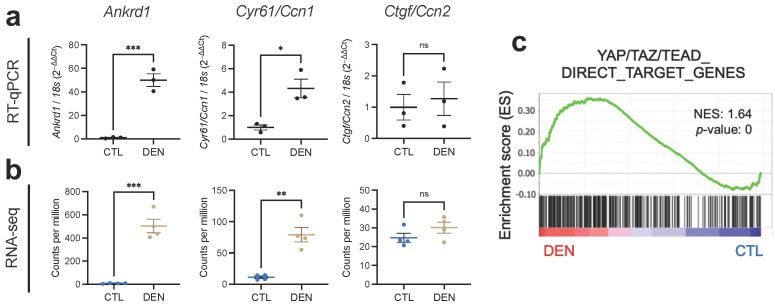
YAP/TAZ/TEAD transcriptional activity is increased in denervated skeletal muscles. (**a**) RT-qPCR analysis of *Ankrd1*, *Cyr61/Ccn1*, and *Ctgf/Ccn2* expression in DEN and CTL (14 days) soleus muscles. N = 3 independent mice. * = *p* < 0.05, *** = *p* < 0.001, ns = not significant; unpaired *t* test. (**b**) RNA-seq analysis (counts per million of mapped reads) of *Ankrd1*, *Cyr61/Ccn1*, and *Ctgf/Ccn2* expressions from DEN and CTL GST muscles. N = 4 independent mice. ** = *p* < 0.01, *** = *p* < 0.001, ns = not significant; unpaired *t* test. (**c**) Gene set enrichment analysis of YAP/TAZ/TEAD direct target genes in DEN and CTL GST muscles. NES = normalized enrichment score.

To assess whether increased expression of YAP/TAZ translates into increased transcriptional activity, we evaluated the gene expression of classical YAP/TAZ target genes, i.e., *Ankrd1*, *Cyr61/Ccn1*, and *Ctgf/Ccn2*. Fourteen days post-denervation, denervated muscles exhibited increased expression levels of *Ankrd1* and *Cyr61/Ccn1* compared to control muscles analyzed by RT-qPCR (Figure 3a). We did not find any changes in *Ctgf/Ccn2* mRNA levels. Again, we complemented our RT-qPCR results with the RNA-seq data and found the same results (Figure 3b). Nevertheless, previous results from our lab showed increased CTGF/CCN2 protein levels, suggesting an earlier transcriptional upregulation [17]. These initial observations suggest that denervation triggers an active state of YAP/TAZ. Thus, we next decided to model transcriptional activity by gene set enrichment analysis (GSEA) using a defined set of YAP/TAZ/TEAD target genes previously reported [39]. GSEA works by ranking whole transcriptomic data in an ordered gene list based on differential expression analysis, from upregulated in denervation (DEN; red color) to upregulated in control (CTL; blue color) (bottom colored bar). Individual genes from the YAP/TAZ signature are scanned throughout the ranked list, and their positions are annotated (black lines above the colored bar). This analysis revealed that most of the target genes of YAP/TAZ fall within the upregulated genes of a denervated muscle obtaining a positive enrichment score (NES = 1.64, nominal *p* = 0), thus indicating increased transcriptional activity of YAP/TAZ under denervation (Figure 3c).

### 2.3. Expanded FAPs Accumulate YAP upon Denervation

**Figure 4:**
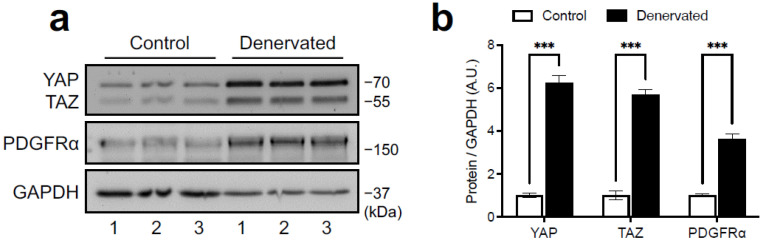

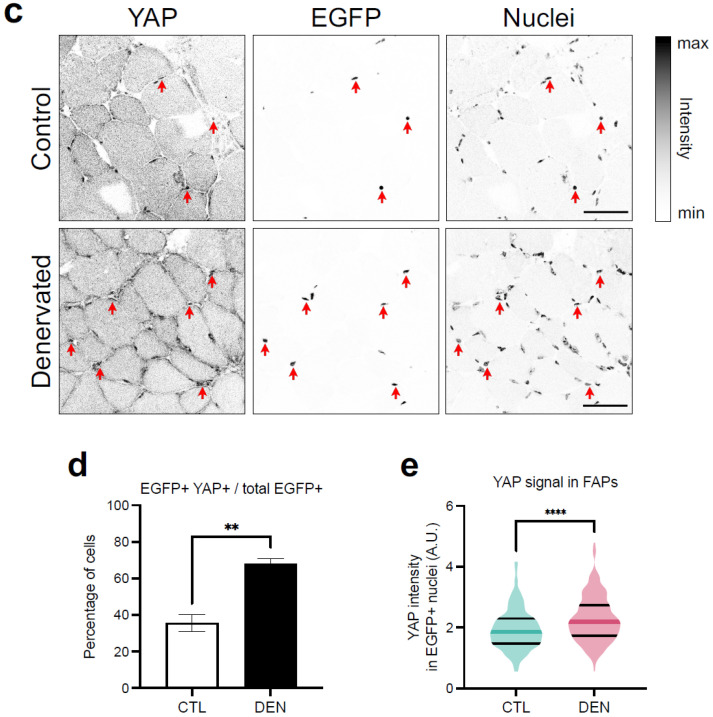
Denervation induces YAP accumulation in FAPs. (**a**) Western blot showing YAP/TAZ and PDGFRα protein expressions in denervated and control TA muscles from the *Pdgfra*^H2B:EGFP/+^ mice. PDGFRα and GAPDH expressions were used as a FAPs marker and loading control, respectively. The numbers at the bottom indicate each independent mouse. (**b**) Densitometric analysis of YAP/TAZ and PDGFRα signals normalized to GAPDH. A.U. = arbitrary units. N = 3 independent mice. *** = *p* < 0.001; unpaired *t* test. (**c**) Representative section of TA showing YAP, EGFP, and total nuclei signals in muscles as in (**a**). Red arrows indicate YAP and EGFP overlay. Scale bar = 50 μm. (**d**) Quantification of the percentage of FAPs (EGFP positive nuclei) expressing YAP (YAP positive and EGFP positive nuclei). (**e**) Quantification of YAP signal intensity in FAPs. A.U. = arbitrary units. N = 3 independent mice. ** = *p* < 0.01, **** = *p* < 0.0001; unpaired *t* test.

The development of fibrosis triggered by denervation correlates with FAPs expansion [8,16,40]. To reveal whether muscular FAPs show regulation of YAP/TAZ upon denervation, we decided to use the transgenic knock-in mouse *Pdgfra*^H2B:EGFP/+^, which expresses the H2B:EGFP fusion protein in cells with an active *Pdgfra* promoter, allowing us to specifically target FAPs in the skeletal muscle [6,40,41]. This mouse strain exhibits atrophy, fibrosis, FAPs accumulation, and induction of *Ankrd1* and *Cyr61/Ccn1* triggered by denervation similar to wild-type mice (Supplementary Figure S1). Western blot analysis of denervated muscle from *Pdgfra*^H2B:EGFP/+^ mice shows that YAP/TAZ were also induced in this genetic model following denervation, indicating that modification of the *Pdgfra* locus does not impact YAP/TAZ induction (Figure 4a). Thus, we carried out immunostaining of YAP in the *Pdgfra*^H2B:EGFP/+^ mice and found that although YAP is basally expressed in the nucleus of a fraction of FAPs in control muscles, denervation significantly increases the number of FAPs expressing YAP, as well as its abundance within the nuclei measured by signal intensity (Figure 4b,c). Thus, part of the effect of YAP/TAZ induction seen at the whole muscle level could be explained by its accumulation in expanded FAPs.

### 2.4. Single-Cell Transcriptomics of Denervated Muscles Reveal Increased YAP/TAZ Activity in FAPs

**Figure 5:**
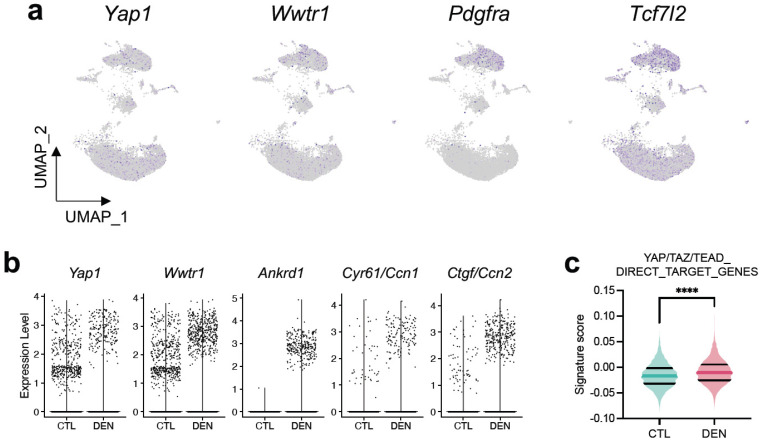
snRNA-seq reveals muscle denervation is coupled with YAP/TAZ activation in FAPs. (**a**) Dimensionality reduction plot (UMAP) showing *Yap1*, *Wwtr1*, *Pdgfra*, and *Tcf7l2* expressions from control skeletal muscles. (**b**) Violin plot showing *Yap1*, *Wwtr1*, *Ankrd1*, *Cyr61/Ccn1*, and *Ctgf/Ccn2* expressions in FAPs from DEN (N = 3634 cells) and CTL (N = 3596 cells) GST muscles. (**c**) Signature score of YAP/TAZ/TEAD direct target genes in FAPs as in (**b**). **** = *p* < 0.0001; unpaired *t* test.

Our previous in vivo findings showing YAP/TAZ induction in FAPs motivated us to investigate whether this accumulation correlates with transcriptional activation of the YAP/TAZ system in FAPs during denervation. To address this inquiry, we analyzed the gene expression profile of FAPs using publicly available single-nucleus RNA sequencing (snRNA-seq) data of whole GST muscle from control and denervated limbs for 14 days [42]. Exploration of gene expression levels at single-nucleus resolution reveals that YAP and TAZ are expressed across nearly every cluster and in the one identified as FAPs (*Pdgfra*+/*Tcf7l2*+) (Figure 5a). Surprisingly, when comparing gene expression of YAP and TAZ in FAPs from control and denervated muscles, we found a reduction in the number of FAPs expressing high levels of YAP and TAZ (Figure 5b; Expression Level ≥ 1). This result contrasts with the accumulation at the protein level and suggests differential regulation of mRNA export from the nucleus or increased YAP/TAZ proteostasis. Nevertheless, gene expression of *Ankrd1*, *Cyr61/Ccn1*, and *Ctgf/Ccn2* demonstrates a considerable number of FAPs expressing high levels of the three target genes during denervation (Figure 5b). We analyzed the enrichment score of the same gene set (YAP/TAZ/TEAD_DIRECT_TARGET_GENES) evaluated in the previous whole muscle analysis (Figure 2c). Figure 5c shows that FAPs from denervated muscle have an increased transcriptional signature of YAP/TAZ compared to FAPs from control muscles, consistent with our results of protein accumulation in these cells. In summary, all our findings, from in vivo to in silico, indicate that FAPs are a novel cell type activating YAP/TAZ in denervated muscle and suggest a potential role of these transcriptional regulators during FAPs activation.

## 3. Discussion

In this work, we studied the role of YAP/TAZ under a neurodegenerative experimental model that leads to the establishment of skeletal muscle fibrosis. Our results prove the involvement of this signaling pathway and assess for the first time its activation in skeletal muscle FAPs.

Here, in the muscle as a whole, we showed that the transcriptional coregulators YAP/TAZ are induced after sciatic nerve transection, suggesting their participation in the development of the fibrogenic process. To the best of our knowledge, we are the first to show that TAZ, and not only YAP [37], potentially plays a role in skeletal muscle fibrosis triggered by denervation. Importantly, the ability of YAP/TAZ to drive fibrosis in other organs indicates that YAP/TAZ activation is a hallmark feature of the fibrotic process possibly conserved in all tissues. For instance, in mice, administration of a known YAP/TAZ-TEAD complex inhibitor, verteporfin (VP) [43], decreased CTGF/CCN2 and collagen I expression in fibrotic kidneys [18,21]. Moreover, TAZ-hemizygous mice resist fibrotic onset triggered by bleomycin in the lungs [44]. In addition, the induction of liver fibrosis by carbon tetrachloride confirms the protective effect of YAP/TAZ inhibition with VP [20]. Whether the skeletal muscle responds similarly to YAP/TAZ inhibition in a fibrotic context requires further research. Judson and colleagues (2013) have already demonstrated that myofiber-specific overexpression of a constitutively active form of YAP (resistant to Hippo-dependent inhibition) in wild-type mice induces features of muscular dystrophy, such as myofiber necrosis, regeneration, and tissue degeneration [45]. However, contrary to this idea, decreased expression of YAP is evident in muscle samples from patients affected by Duchenne muscular dystrophy [46].

At the molecular level, the capacity of YAP/TAZ to regulate fibrogenic processes could be attributed to their participation as molecular mediators of the differentiation of fibroblasts/stromal cells into myofibroblasts. Our results show that FAPs, characterized as multipotent stromal cells, activate YAP/TAZ during denervation, suggesting a role in their activation, proliferation, and/or differentiation. YAP/TAZ activation could be explained by the fact that these transcriptional coregulators act as nodes of several signaling pathways associated with fibrosis [25]. Among the factors that stimulate the differentiation of fibroblasts derived from diverse tissues, the most studied are those related to TGF-β1 signaling, whose effects can interestingly be prevented through YAP/TAZ inhibition [21,47,48,49]. We have previously shown that TGF-β1 signaling is upregulated during denervation and in a neurodegenerative genetic model of ALS [15,17]. TGF-β1-SMAD and YAP/TAZ pathways regulate multiple common highly inducible target genes, such as *Ctgf/Ccn2* or *Cyr61/Ccn1*; thus, cooperative action between these pathways is suggested to be at the transcriptional level through the assembly of a large transcriptional complex. In the skeletal muscle, *Ctgf/Ccn2* acts as a pro-fibrotic factor [50,51]. Overexpression of *Ctgf/Ccn2* in healthy muscle by adenovirus infection induces tissue degeneration and muscle fibrosis, leading to reduced muscle functionality [50]. Moreover, blockade or decreased expression of *Ctgf/Ccn2* in skeletal muscle fibrotic models results in reduced tissue fibrosis [15,17,51]. Our snRNA-seq analysis reveals that after denervation, FAPs upregulate the expression of YAP/TAZ target genes, including *Ctgf/Ccn2* (Figure 5b). These observations suggest a possible mechanism where TGF-β1 signaling in FAPs activates YAP/TAZ, which, coupled with SMAD mediators, induces *Ctgf/Ccn2* to control ECM remodeling and fibrotic development.

Another possible mechanism of YAP/TAZ activation in FAPs during the fibrotic stage of denervation may be due to increased ECM stiffness. Indeed, cellular responses to matrix stiffness have been widely reported as critical inductors of tissue fibrosis [52]. Other groups have demonstrated increased YAP/TAZ signals in cells that reside and accumulate in high-stiffness zones in fibrotic tissues [18,19,47]. Fibrotic skeletal muscle ECM exhibits high stiffness [53,54,55]. Recently, the pro-fibrotic response of FAPs to ECM stiffness was reported for the first time [56]. In response to biomimetic substrates of high stiffness, FAPs display nuclear accumulation of YAP and induction of myofibroblast differentiation, which can be prevented by VP addition. This demonstrates the conserved ability of FAPs to adopt a pro-fibrotic phenotype in response to ECM stiffness.

Adding more complexity, YAP/TAZ have also been shown to participate in the Wnt/β-catenin signaling, intimately related to the destruction complex of β-catenin [28,29]. Activation of the pathway induces the release of β-catenin and YAP/TAZ from the destruction complex allowing β-catenin/TCF/LEF and YAP/TAZ/TEAD transcriptional activities. Interestingly, the Wnt3a ligand is upregulated in denervated muscles [57]. Whether FAPs induce YAP/TAZ signaling by a mechanism dependent on Wnt/β-catenin remains unexplored; however, FAPs are known to express high levels of the canonical Wnt/β-catenin transcription factor *Tcf7l2* (Figure 5a) [8,9,11,12], suggesting a possible target cell responding to high levels of Wnt ligands during denervation.

In conclusion, this work is the first to reveal the activation of YAP/TAZ in FAPs associated with skeletal muscle fibrosis in vivo and with single-nucleus resolution. We believe that our results could establish a starting point to assess the functional role of YAP/TAZ in the fibrogenic process of the skeletal muscle and the cell fate decisions of FAPs, resulting in a better understanding of FAPs biology and fibrosis.

## 4. Materials and Methods

### 4.1. Animal Experiments

Animal experiments were performed with approval and in accordance with the Animal Ethics Committee of Pontificia Universidad Católica de Chile (Protocol 220427014). Mice were maintained before and during experiments in a 12 h light-dark cycle with access to food and water. Limb muscle denervation was conducted in 5–6 months old C57BL/10 wild-type or Pdgfra^tm11(EGFP)Sor^ (JAX stock #007669; [41]) mice as previously described [17]. Briefly, 2–5 mm of the sciatic nerve was transected at the gluteal and biceps femoris muscles level of the left hindlimb, whereas right-side muscles without surgery were used as controls. Fourteen days post-surgery, animals were sacrificed. TA, GST, or soleus muscle from denervated and control hindlimbs were collected, frozen in liquid nitrogen (plus isopentane for cryosectioning) and stored at −80 °C until processing.

### 4.2. Protein Extraction and Western Blot

Protein extraction, SDS-PAGE, and Western blot were performed as previously described [6]. Briefly, 40–60 μg of protein was separated by SDS-PAGE, electrophoretically transferred to PVDF membranes, blocked with 5% milk in TBS (50 mM Tris-HCl, pH 7.6; 150 mM NaCl), and probed overnight at 4 °C with the following antibodies: anti-YAP/TAZ (8418, Cell Signaling, Danvers, MA, USA), anti-Fibronectin (F3648, Sigma-Aldrich, St. Louis, MO, USA), and anti-GAPDH (sc-365062, Santa Cruz Biotechnology, Santa Cruz, CA, USA). To detect the primary antibody, horseradish-peroxidase-conjugated secondary antibodies and SuperSignal™ Luminol/Enhancer substrates (Thermo, Waltham, MA, USA) were used to generate chemiluminescence. Protein expression, measured as absolute pixel density, was determined using ImageJ software (v.1.53k, NIH, Bethesda, MD, USA).

### 4.3. RNA Isolation and RT-qPCR

RNA extraction from the soleus muscle was performed using TRIzol (Invitrogen, Carlsbad, CA, USA) according to the manufacturer’s instructions. One μg of isolated total RNA was reverse-transcribed into complementary DNA using random primers and M-MLV reverse transcriptase (Invitrogen, Carlsbad, CA, USA). RT-qPCR was performed in triplicate on an Eco Real-Time PCR System (Illumina, San Diego, CA, USA) using PowerUp™ SYBR™ Green Master Mix (Applied Biosystems, Foster City, CA, USA) and primer sets for mouse *Yap1* (F: 5′-GGA AGG AGA AGC AAT GAA CAT AGA-3′ and R: 5′-CGT CCA AGA TTT CGG AAC TCA-3′), *Wwtr1* (F: 5′-CTT GCT GGT GTT GTT GAT TC-3′ and R: 5′-ATC AGC CTC TGA ATC ATG TGA A-3′), *Ankrd1* (F: 5′-GGA TGT GCC GAG GTT TCT GAA-3′ and R: 5′-GTC CGT TTA TAC TCA CAG AC-3′), *Cyr61/Ccn1* (F: 5′-TAA GGT CTG CGC TAA ACA ACT C-3′ and R: 5′-CAG ATC CCT TTC AGA GCG GT-3′), *Ctgf/Ccn2* (F: 5′-CAG GCT GGA GAA GCA GAG TCG T-3′ and R: 5′-CTG GTG CAG CCA GAA AGC TCA A-3′), and for reference mouse *Gapdh* (F: 5′-TGA TGA CAT CAA GAA GGT GGT GAA G-3′ and R: 5′-TCC TTG GAG GCC ATG TAG GCC AT-3′) or *18s* (F: 5′-TGA CGG AAG GGC ACC ACC AG-3′ and R: 5′-GTT TGC GAT GGT ACA GCT TAT TC-3′) at a final concentration of 300 nM (Integrated DNA Technologies, Coralville, IA, USA). mRNA expression was determined using the comparative 2^−ΔΔCt^ method and expressed as fold-changes relative to control muscles.

### 4.4. Immunofluorescence

Tissue sectioning and immunofluorescence were performed as previously described [6]. Samples were incubated with anti-YAP (1:100, sc-376830, Santa Cruz Biotechnology, Santa Cruz, CA, USA) overnight at 4 °C. To prevent endogenous mouse IgG detection, Ready Probes^TM^ Mouse on Mouse IgG Blocking Solution (Invitrogen, Carlsbad, CA, USA) was used according to the manual’s instructions and before primary antibody incubation. The primary antibody was detected by incubation with a secondary antibody Alexa Fluor^®^ 568 goat anti-mouse IgG (H + L) (1:500, A11004, Invitrogen, Carlsbad, CA, USA). Samples were stained for total nuclei with Hoechst 33342 (2 mg/mL) for 10 min at RT and mounted with a fluorescent mounting medium (Dako, Glostrup, Denmark). Fluorescent images were acquired using LSM 880 ZEISS microscope with Airyscan detector mounted with an LD LCI Plan-Apochromat 40× objective (NA = 1.20). Images were presented as inverted grayscale. Analysis of images in Figure 4d,e was performed using ImageJ software (v.1.53k, NIH, Bethesda, MD, USA). Briefly, to determine the number of YAP+ and EGFP+ cells, the image of the YAP channel was converted to 8-bit format and segmented (threshold = MaxEntropy dark) to obtain only YAP-positive areas above the defined threshold. The resulting binary image was analyzed for colocalization with EGFP+ cells, and the number of double positive cells (YAP+ and EGFP+) was counted. For YAP signal intensity, the EGFP channel was converted to 8-bit format, blurred with a Gaussian filter (sigma = 2.0), segmented (threshold = MaxEntropy dark), analyzed for particles (size = 2.0), and added to ROI Manager. YAP channel intensity was measured within every segmented EGFP+ nuclei and normalized to the whole image.

### 4.5. Transcriptomics Analyses

Bulk RNA-seq data from control and denervated muscles were downloaded from NBCI Sequenced Read Archive (SRA) under accession code SRP196460 [38] (samples: CTL_1: SRR9026466; CTL_2: SRR9026467; CTL_3: SRR9026508; CTL_4: SRR9026456; DEN_1: SRR9026506; DEN_2: SRR9026501; DEN_3: SRR9026504; DEN_4: SRR9026499). The high quality of sequenced libraries was determined using the FastQC package (v.0.11.9). Reads were aligned into the mouse reference genome assembly GRCm39 using Hisat2 with default parameters (v.2.2.1). Mapped read files were converted to reads per annotated gene counts using version 108 of GRCm39 for transcriptome annotation and processed in R for Mac OS X GUI (v.4.2.1), RStudio (v.2022.07.1), and the R package Rsubread (v.2.12.0). Raw counts were processed for normalization using the Trimmed mean of M-values (TMM) methods in the R package edgeR (v.3.40.0). GSEA of YAP/TAZ signature was analyzed using a custom-built gene set of previously documented YAP/TAZ target genes [39] and the desktop version of GSEA (v.4.3.2 Mac App).

Single-nucleus transcriptomics of denervated and control muscles was obtained from Gene Expression Omnibus (GEO) GSE183802 [42] (supplementary file: GSE183802_snRNA-seq_data_CTL_and_denervation.rds.gz). Processed data were analyzed in R and with the Seurat package (v.4.2.1). AddModuleScore function was used to assign YAP/TAZ signature score to the Seurat object.

### 4.6. Statistical Analysis

The number of replicates used for each experiment is indicated in the figure legends. Data are presented as the mean ± SEM. Statistical significance between two groups was determined by unpaired Student’s t-test using Prism 9 for macOS (v. 9.5.0, Graphpad Software Inc., Boston, MA, USA). Differences were considered statistically significant with a *p* < 0.05.

## Data Availability

Publicly available datasets were analyzed in this study. This data can be found here: https://www.ncbi.nlm.nih.gov/geo/query/acc.cgi?acc=GSE183802 (accessed on 17 December 2022) and https://www.ncbi.nlm.nih.gov/sra/SRP196460 (accessed on 1 December 2022).

## Supplementary Materials

The following supporting information can be downloaded at: https://www.mdpi.com/article/10.3390/ijms24065585/s1.
