# Supplementary material for: Denervation Drives YAP/TAZ Activation in Muscular Fibro/Adipogenic Progenitors
Source: Int J Mol Sci. 2023 Mar 15;24(6):5585. doi: 10.3390/ijms24065585 (PMC10059792; doi:10.3390/ijms24065585)
Supplement: Supplementary file 1 [file ijms-24-05585-s001.zip › ijms-2229932-supplementary.pdf]

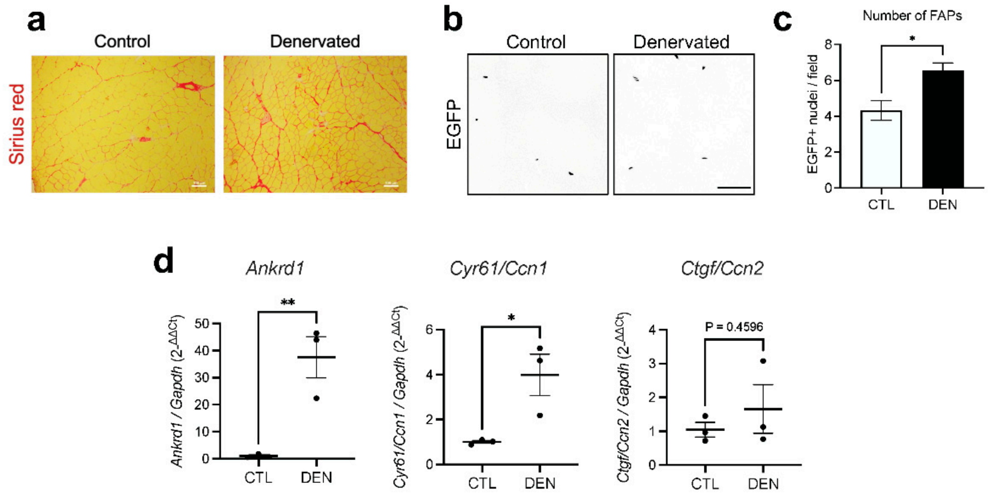


**Figure S1.** Denervation in the *Pdgfra*$^{H2B:EGFP/+}$ mice resulted in atrophy, fibrosis, FAPs accumulation, and YAP/TAZ target gene induction. (**a**) Sirius red staining of denervated and control TA muscle sections from the *Pdgfra*$^{H2B:EGFP/+}$ mice. Scale bar = 100 µm. (**b**) EGFP signal from muscle sections as in a. Scale bar = 50 µm. (**c**) Quantification of EGFP positive nuclei per field. N=3 independent mice. * = $p < 0.05$; unpaired t test. (**d**) RT-qPCR analysis of *Ankrd1*, *Cyr61/Ccn1*, and *Ctgf/Ccn2* expression in DEN and CTL muscles as in a. N = 3 independent mice. * = $p < 0.05$, ** = $p < 0.01$; unpaired t test.
